# MicroRNAs and lncRNAs in senescence: A re‐view

**DOI:** 10.1002/iub.1373

**Published:** 2015-05-19

**Authors:** Oliver Bischof, Ricardo Iván Martínez‐Zamudio

**Affiliations:** ^1^ Institut Pasteur, Laboratory of Nuclear Organization and OncogenesisDepartment of Cell Biology and InfectionParisFrance; ^2^ INSERMU993ParisFrance

**Keywords:** aging, senescence, transcription factors, transcriptional regulation, noncoding RNA, chromatin, RNA interference

## Abstract

Cellular senescence is a stress response to a variety of extrinsic and intrinsic insults that cause genomic or epigenomic perturbations. It is now widely recognized as a potent tumor suppressor mechanism as well as a biological process impacting aging and organismal development. Like other cell fate decisions, senescence is executed and maintained by an intricate network of transcription factors (TFs), chromatin modifiers, and noncoding RNAs (ncRNAs). Altogether, these factors cooperate to implement the gene expression program that initiates and sustains the senescent phenotype. In the context of senescence, microRNAs (miRs) and long ncRNAs have been found to play regulatory roles at both the transcriptional and post‐transcriptional levels. In this review, we discuss recent developments in the field and point toward future research directions to gain a better understanding of ncRNAs in senescence. © 2015 IUBMB Life, 67(4):255–267, 2015

## Introduction

**Figure 1:**
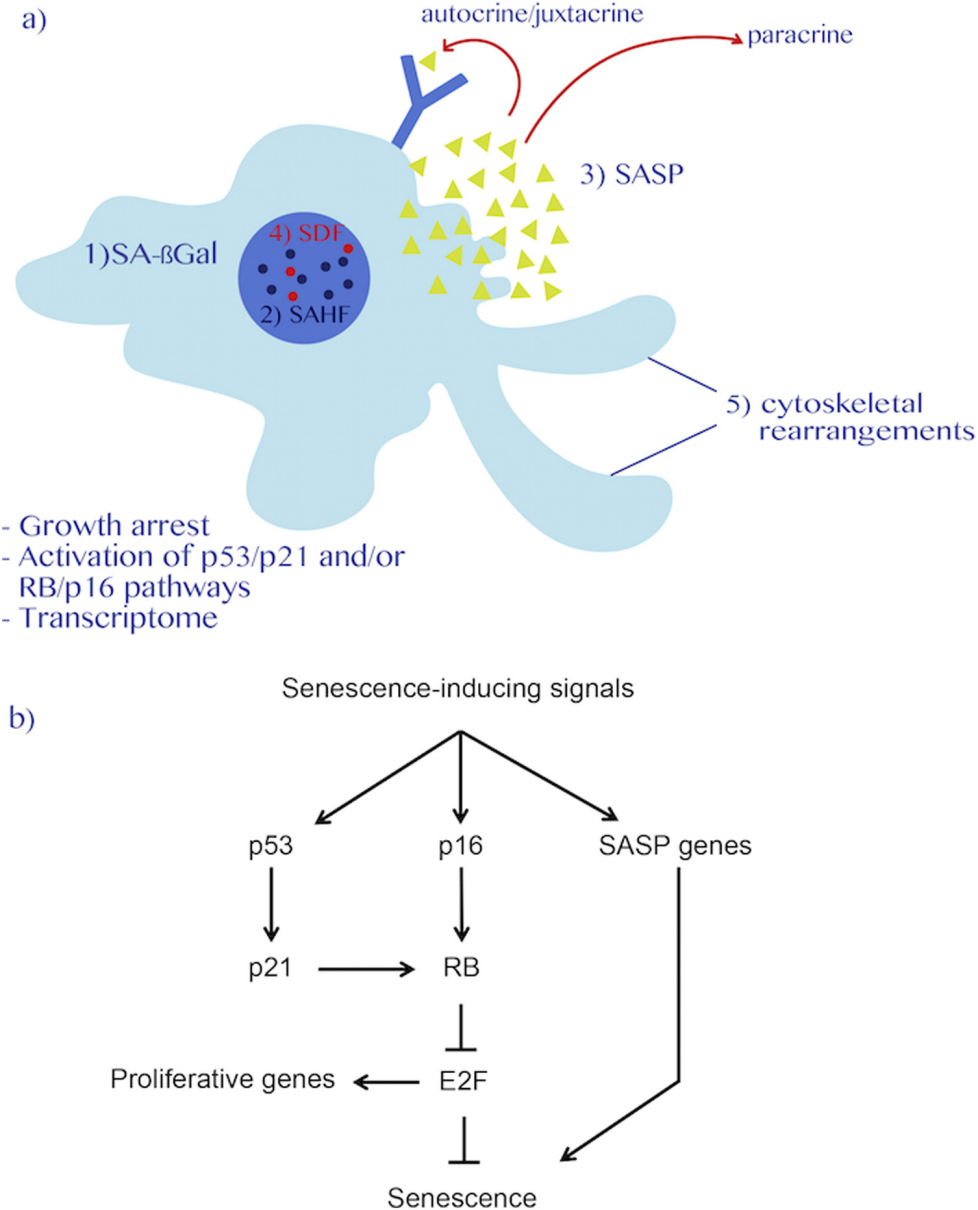
Biomarkers of senescence. (a) Senescence is a stable growth arrest that is orchestrated by the p53/p21 and/or RB/p16 tumor suppressor pathways. Biomarkers of the senescence phenotype may include: 1) increased senescence‐associated β‐galactosidase activity (SA‐βGal); 2) reorganization of chromatin architecture as exemplified by senescence‐associated heterochromatic foci (SAHF) and reflected by a dramatic change in gene expression profile; 3) acquisition of a senescence‐associated secretory phenotype (SASP) that can enforce senescence in autocrine/juxtacrine and paracrine fashion (arrows); 4) constitutive senescence‐associated DNA damage foci (SDF); and 5) extensive cytoskeletal rearrangements characterized by a flat cell morphology. (b) Schematic representation of the two major senescence‐effector pathways (*i.e*., p53/p21 and RB/p16). Several senescence‐inducing stimuli such as hyperactivated oncogenes, DNA damage, cytokines, and therapeutic agents can activate these pathways to induce senescence.

**Figure 2:**
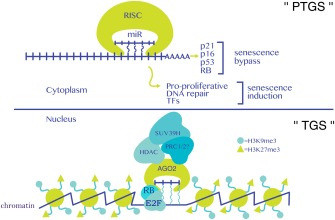
Functions of miRs in senescence. In the cytoplasm (top half of figure), canonical post‐transcriptional gene silencing (PTGS), involving translational suppression and transcript degradation, modulates senescence. Senescence bypass is often observed when miRs affect mRNAs coding for prosenescence factors like p21, p16, p53, or RB, whereas senescence induction may be achieved by targeting mRNAs coding for pro‐proliferative, DNA repair, and transcription factor (TF) genes. A miR/AGO2 containing corepressor complex facilitates the onset of senescence via a noncanonical nuclear function by recruiting RB and chromatin‐modifying complexes to pro‐proliferation E2F target genes (bottom half of figure). This process installs a repressive chromatin environment (*i.e*., H3K9me3 and H3K27me3) and engages transcriptional gene silencing (TGS). The presence of histone‐modifying enzymes SUV39H, PRC1, and PRC2 at these promoters is assumed and needs to be experimentally established. Wavy blue lines indicate hybridization between a miR and a putative target promoter RNA and/or target promoter DNA.

Cellular senescence, as first described by Hayflick and Moorhead 1, referred to the finite lifespan of primary human fibroblasts in culture and was quickly stipulated to be a causal factor of aging. Today, we know that these observations describe only one particular type of cellular senescence that is caused by the attrition of telomeres (*i.e*., replicative senescence [RS]), a naturally occurring process that takes place every cell division 2. Our current expanded view of cellular senescence encompasses numerous physiological processes such as tumor suppression, aging, age‐related diseases, and regulation of development 3, 4, 5. Senescence can be triggered by a variety of extrinsic and intrinsic insults that ultimately cause genomic or epigenomic perturbations. These insults include, for example, supraphysiological oncogenic signaling (*e.g*., oncogenic RAS, CCNE1, collectively referred to as oncogene‐induced senescence [OIS]), telomere dysfunction, reactive oxygen species (ROS), cytokines, and other toxins/stressors including anticancer treatments (therapy‐induced senescence [TIS]). Irrespective of the stimulus, the senescence program is executed on persistent activation of the p53/p21 (*alias* CDKN1A) and/or RB (retinoblastoma)/p16 (*alias* CDKN2A; p16INK4a) tumor suppressor pathways, which, by‐and‐large, orchestrate the transition to and maintenance of the senescent phenotype 3 (Figure 1). p53 and RB are transcriptional regulators that are frequently rendered dysfunctional in cancerous cells and are the archetypical tumor suppressor proteins in mammalian cells 6. The growth‐inhibitory activity of the two proteins is mainly regulated by post‐translational modifications, including phosphorylation, acetylation, ubiquitination, and sumoylation 7. P53 directly modulates p21 gene expression; however, the mechanism that regulates p16 expression is, to date, incompletely understood although it involves polycomb group proteins EZH2, BMI1, CBX7 as well as TF ETS2, noncoding RNAs (ncRNAs), and general chromatin architectural changes 8, 9. Ultimately, p21 and p16 transform hyperphosphorylated, inactive RB to its hypophosphorylated, active state. Active RB specifically represses transcription of genes that drive cell cycle progression such as PCNA, CCNA, and CCNB due to the inhibition of E2F family (E2Fs 1–8) of transcription factors (TFs) by recruiting a corepressor complex involving histone deacetylases (HDACs), histone methyltransferases (HMTs), and the RNA interference (RNAi) machinery at their respective promoters 10 (Figure 2).

The senescent phenotype is variably characterized by a number of biomarkers including salient chromatin structure, differential DNA methylome, gene expression, metabolic, and cytomorphological changes as well as a stable cell cycle arrest, and chronic DNA damage response (DDR) among others; more specifically, these changes include induction of senescence‐associated beta galactosidase (SA‐βGal) activity, formation of senescence‐associated heterochromatin (SAHF), and DNA damage foci (SDF), a flat cell morphology and secretion of a variety of growth factors, metalloproteinases, and inflammatory cytokines, collectively known as the senescence‐associated secretory phenotype (SASP; Fig. 1; ref. 3). The SASP is driven by a persistent DDR and influences multiple biological processes ranging from stimulation of cell proliferation 11, reinforcement of the senescent phenotype 12, facilitating cell type transitions 13, disrupting stem cell niche homeostasis 14, aiding optimal wound healing 15, and promoting the immune clearance of senescent cells 16, 17, 18. Like any other cell fate, the instigation and maintenance of the senescent phenotype is controlled by an intricate network of TFs, chromatin modifiers, and ncRNAs, which themselves dynamically feedback to senescence‐inducing stimuli. Altogether, these factors work in concert to implement the gene expression program that initiates and sustains the senescent phenotype.

The regulatory role of ncRNAs in cell fate determination and maintenance has become increasingly appreciated, with novel classes of regulatory RNAs being discovered in the recent years. This has been fuelled by the development of powerful next‐generation sequencing technologies that have not only facilitated the discovery of novel regulatory RNA molecules 19 but also, and more importantly, have brought to light that the presence of ncRNA in cells is pervasive as a result of high rates of noncoding transcription, which account for up to 80% of total cellular transcription 20. Indeed, the RNAi machinery including short‐interfering RNAs (siRNAs) and microRNAs (miRs), long ncRNAs (lncRNAs), piwi‐associated small RNAs (piRNAs), enhancer RNAs (eRNAs), circular RNAs (cRNAs), DDRNAs/diRNAs, and splice site RNAs (spliRNAs) are only some of the ncRNAs that have been found to modulate gene expression and genomic stability, highlighting the critical regulatory role of ncRNAs in cellular processes 19, 21, 22. With regard to cellular senescence, to date, miRs and lncRNAs were found to have important regulatory roles at several levels including (i) alteration of gene expression at the transcriptional and post‐transcriptional levels and (ii) maintaining genome stability and facilitating DNA repair. In this review, we will provide the present state of knowledge of the role of miRs and lncRNAs in senescence and offer perspectives for future research.

## The RNAi Machinery and MiRs in the Modulation of Senescence

Historically, miRs were first described to play regulatory roles in the development of *Caenorhabditis elegans* 23. MiRs are a class of small RNAs of ∼22 nucleotides that silence gene expression predominantly through a post‐transcriptional mechanism (post‐transcriptional gene silencing [PTGS]) that entails translational repression and transcript decay 24. Their biosynthesis involves a multistage process that is dependent on the RNAi machinery: (i) RNA Polymerase II (RNA Pol II)‐dependent transcription and polyadenylation of miR genes (which can be up to several kb long), leading to the formation of long pri‐miRs containing stem loop structures, (ii) nuclear cropping of the long pri‐miR by the DGCR8/Drosha heterodimer into smaller 60–100 bp long pre‐miRs, and (iii) the Exportin 5 (XPO5) and RanGTP‐dependent nuclear export of pre‐miRs and their cytoplasmic maturation into ∼22 bp by the RNAse Dicer 25. Subsequently, double‐stranded miRs are loaded onto argonaute (AGO) proteins in a process that involves the selection of the guide strand via the recognition of its 3′ end and the 5′ phosphate by AGOs PAZ and MID domains with the concomitant degradation of the passenger strand, generating the RNA‐induced silencing complex (RISC). The RISC is a negative regulator of gene expression that achieves target specificity by virtue of the guide strand, which binds single‐stranded RNAs via base pairing. This initial binding is mediated by the so‐called seed sequence spanning nucleotides 2–6 of the guide strand. It is thought that the nature of mRNA recognition by the RISC, that is, perfect versus imperfect base pairing, defines the gene silencing mechanism of the target mRNA. When perfect base pairing occurs, target mRNAs are degraded via the endonucleolytic slicer activity of AGO2. In contrast, imperfect base pairing can lead to translational repression of the target mRNA 26. Additional regulatory layers can be achieved by the miR pathway due to the imperfect base‐pairing nature of its targeting mechanism, thus allowing for the regulation of multiple mRNAs by a single miR as well as individual mRNAs by multiple miRs. Apart from its canonical role in PTGS, dedicated roles for miRs in TGS have surfaced recently. Cytonuclear shuttling of AGO proteins and miRs is in part mediated by Importin 8 (IPO8), CRM1, and XPO5 (refs. *27* and *28*). Nuclear miRs have been shown to inhibit pri‐miRs processing 29, modulate the stability of lncRNAs 30, and recruit chromatin‐modifying complexes to target genes 31, 32. Thus, miRs fine‐tune gene expression both at transcriptional and post‐transcriptional levels. A flurry of recent reports highlighted the important modulatory role of the miR pathway in cellular senescence. Similar to their coding counterparts, noncoding miRs may induce, accelerate, bypass, or delay senescence by impacting on the p53/p21 and the RB/p16 pathways.

To identify miRs that modulate senescence, transcriptional profiling by microarrays or RNA‐sequencing (RNA‐seq) has been used primarily. Differentially regulated miRs in senescence are then further characterized by assessing their effects on well‐known senescence biomarkers such as proliferative capacity, induction of SA‐βGal activity, SAHF formation, activation of the p53/p21 and RB/p16 pathways, and modulation of the SASP (Fig. 2). Using this approach, several miRs have been found to promote senescence bypass or induction in different cell types and under different senescence‐inducing stimuli (Table 1).

**Table 1:** miRs and lncRNAs in cellular senescence

MiRs/lncRNA	Cell type/tissue	Animal model	Species	Effect on senescence	Possible target/mechanism	Reference
MiR‐16	Mammary/lung epithelial cells	N/A	Hs	–	N/A	33
MiR‐21	Mammary/lung epithelial cells	N/A	Hs	–	N/A	33
MiR‐382	Mammary/lung epithelial cells	N/A	Hs	–	N/A	33
MiR‐106b	Mammary epithelial cells	N/A	Hs	–	p21 3′UTR	34
MiR‐378a‐5p	Fibroblasts	N/A	Hs	–	Interference with activation of the p16 pathway	35
MiR‐146a/b	HUVECs, fibroblasts	N/A	Hs	–	NOX4a 3′UTR, reduced IRAK expression	36, 58, 60
MiR‐449a	Fibroblasts	ERCC1−/−	Mm	–	N/A	37
MiR‐445	Fibroblasts	ERCC1−/−	Mm	–	N/A	37
MiR‐128	Fibroblasts	ERCC1−/−	Mm	–	N/A	37
MiR‐19b	HeLa, MCF7, and HuH	N/A	Hs	–	Interference with activation of the p53 pathway	38
Dicer	Skin	Dicer conditional; Dicer−/−	Mm	–	N/A	39, 40
DGCR8	Fibroblasts	N/A	Hs; Mm	–	Interference with activation of the p53 pathway	41
MiR‐519	Fibroblasts	N/A	Hs	+	HuH 3′UTR, p53/p21 and Rb/p16 pathway activation, increased SASP	42
MiR‐26b	Mammary epithelial cells	N/A	Hs	+	PRC2 members 3′UTR	44
MiR‐181a	Mammary epithelial cells	N/A	Hs	+	PRC2 members 3′UTR	44
MiR‐210	Mammary epithelial cells	N/A	Hs	+	PRC2 members 3′UTR	44
MiR‐424	Mammary epithelial cells	N/A	Hs	+	PRC2 transcriptional repression	44
MiR‐22	Cardiac fibroblast	N/A	Mm	+	N/A	45
MiR‐24	Cardiac fibroblast	N/A	Mm	+	N/A	45
MiR‐29a/b	Kidney, lung, heart, and liver; skeletal muscle	Kl−/−; N/A	Mm; Rn	+	Type IV collagens 3′UTR; IGF1, p85α, and MYBL2 3′UTRs	46, 47
MiR‐494	Fibroblasts	N/A	Hs	+	HnRNPA3, RAD32B, and SYNCRIP 3′UTRs	49
MiR‐34	RCC; CLL	N/A	Hs	+	NOTCH1, E2F1, CDK6, and SIRT1 3′UTRs; p53 pathway activation	53, 54
MiR‐449	HCC; PCC; LCC	Gastrin−/−, *Helicobacter pylori* infection	Hs; Mm	+	MYC, CDK6, and CCNE2 3′UTRs; CCND1 3′UTR, Rb pathway activation; E2F3 3′UTR	55, 56, 57
MiR‐335	hMSCs	N/A	Hs	+	Activation of p16 pathway	61
MiR‐212	HepG‐2	N/A	Hs	+	Activation of p16 pathway	70
H19/miR‐675	Prostate	Pten prostate conditional deletion	Mm	–	RB 3′UTR	73
let7	Fibroblasts	N/A	Hs	+	Recruitment of AGO2/HDAC complex to E2F target gene promoters	31
ANRIL	Fibroblasts and PCC	N/A	Hs	–	Recruitment of PRC1 to the INK4 locus	8
PANDA	Fibroblasts	N/A	Hs	+/−	Recruitment of SAFA/BMI1 to target promoters/sequestration of NF‐YA	81
HOTAIR	HeLa	N/A	Hs	+	Facilitates ubiquitination of ATXN1 and Snurportin	85
TERRA	HeLa; fibroblasts	N/A	Hs	+/−	Recruitment of shelterin proteins/recruitment of LSD1/MRE11 complex	86, 87, 88, 89

Abbreviations: N/A, not available; Hs, Homo sapiens; Mm, Mus musculus; HUVECs, human umbilical vein endothelial cells; CLL, chronic lymphocytic leukemia; RCC, renal cell carcinoma; LCC, lung cell carcinoma; PCC, prostate cancer cell.

### Senescence‐Obstructing MiRs

In primary human mammary and lung epithelium undergoing senescence induced by ionizing radiation and cisplatin, microarray screenings identified a set of ∼30 cell type‐specific, differentially expressed miRs. Additional analyses of these candidates identified *miR‐16*, *miR‐21*, and *miR‐382* as miRs that, when overexpressed, enhance resistance to doxorubicin and cisplatin treatment in breast and lung cancer cell lines by inhibiting apoptosis and, in the case of *miR‐21*, restoring proliferative capacity and promoting colony formation 33. Moreover, in mammary epithelial cells undergoing OIS, a microarray screen identified the 106b family of miRs as capable of bypassing senescence. The mechanism underlying the senescence likely involves targeting of the 3′ untranslated region (UTR) of *p21*, as suggested by luciferase assays, and this is correlated with a decrease in the nuclear localization of *p21* 34. A similar study found that *miR‐378a‐5p* could promote OIS bypass in human fibroblasts by dampening the activation of the p16 pathway, thus leading to increased proliferation 35. In human umbilical vein endothelial cells, a microarray screening identified *miR‐146a* as a pro‐proliferative miR that prevents senescence by downregulation of the NAPDH oxidase subunit *NOX4* via targeting of its 3′UTR. During RS, *miR‐146a* levels decrease, leading to derepression of *NOX4* and presumably increased ROS generation leading to DNA damage‐induced senescence 36. Similarly, gene expression profiling in embryonic fibroblasts isolated from *ERCC1*‐deficient mice, which undergo premature aging due to the accumulation of DNA damage by oxidative stress, identified *miR‐449a*, *miR‐455*, and *miR‐128* as specifically downregulated, with similar expression patterns for these miRs in the kidneys of elderly mice 37. However, whether overexpression of these miRs could reverse features of the senescent phenotype was not tested. Using a novel dual‐color fluorescence microscopy screen based on the colocalization of red fluorescent protein (RFP)‐tagged miR constructs with a green fluorescent protein (GFP)‐tagged 3′UTR region of *p53*, the microRNA *miR‐19b* was found to target *p53* in multiple cancer cell lines (*i.e*., HeLa, MCF7, and HuH). Overexpression of *miR‐19b* in these cell lines reduces the expression of p53, p21, and BAX at the protein level and prevents senescence. Importantly, the senescence‐bypassing effects of *miR‐19b* can be reversed by overexpression of a miR‐19b‐specific “RNA sponge.” When injected in nude mice, miR‐19b‐overexpressing cancer cell lines promote tumor growth and metastases 38. Thus, *miR‐19b* may aid in bypassing senescence by dampening a p53‐dependent senescence response.

Further support for a role of the miR pathway in the maintenance of the proliferative state has come from animal studies. Indeed, embryonic fibroblasts isolated from Dicer‐conditional knockout mice display features of premature senescence in the developing limb and in adult skin characterized by activation of the p53 and INK4a pathways 39, 40. Accordingly, deletion of the p53 or INK4a/ARF (p19^ARF^) locus rescues cells from premature senescence induced by Dicer ablation 39, 40. A similar phenotype is observed when the Drosha‐auxiliary protein DGCR8 is downregulated in both mice and human primary fibroblasts, which undergo p53‐dependent premature senescence 41. Although these studies clearly support a general role for the miR pathway in the maintenance of the proliferative state, it is unclear if the effects described therein are specific to a subset of miRs or if it is a built‐in cellular response to the disruption of the miR pathway.

### Senescence‐Promoting MiRs

In a human fibroblast RS model, a microarray‐based screen identified *miR‐519* as strongly upregulated in senescent cells. When overexpressed in proliferating fibroblasts, *miR‐519* induced a stable growth arrest that is accompanied by secretion of SASP factors IL6 and IL8 and activation of both the p53/p21 and the RB/p16 tumor suppressor pathways. Mechanistically, *miR‐519* appears to promote senescence by targeting the 3′UTR of the RNA‐binding protein HuR 42, which is involved in mRNA stabilization through binding to AU‐rich elements (AREs; ref. 43). Using a functional miR screen to identify modulators of p16‐induced senescence, a set of four senescence‐associated miRs (SA‐miRs; *miRs‐26b*, *181a*, *210*, and *424*) was found to drive senescence by the transcriptional repression of polycomb repressor complex (PRC) 2 members 44. Interestingly, when individually overexpressed, SA‐miR‐mediated PRC2 transcriptional repression enhances senescence by derepression of other SA‐miRs, thus suggesting a feedback loop mechanism that maintains the balance between the expression of SA‐miRs and PRC2 members. An additional layer to this mechanism is added by the necessity for an intact p16 pathway, as downregulation of p16 in these cells both reduces SA‐miRs and increases the expression of PRC2 components 44. During the onset of heart fibrosis in aging mice, a differential miR screen identified miRs *miR‐22* and *miR‐24* as upregulated in an age‐dependent manner. Overexpression of these miRs in neonatal cardiac fibroblasts induced SA‐βGal activity, decreased proliferative potential, and increased chemotaxis 45. Based on these findings, the authors proposed that *miR‐22* and *miR‐24* may play a role in the age‐dependent fibrosis of cardiac tissue 45. Several screening studies have identified *miR‐29a/b* as upregulated in several senescence models. In a mouse model of progeria (klotho‐deficient mice), a miR screen identified *miR‐29a/b* as upregulated in various tissues including kidney, lung, heart, and liver, a pattern that is also observed in elderly mice 46. At a molecular level, *miR‐29a/b* targets type IV collagen genes in proliferating cells and young mice, leading to reductions of type IV collagens in the abovementioned tissues. However, the contribution of the *miR‐29a/b*‐dependent loss of type IV collagen to senescence in these tissues has not been assessed. The degenerative loss of skeletal muscle mass, known as sarcopenia, occurs naturally during aging. In a rat model of age‐induced sarcopenia, *miR‐29a/b* was found to be differentially upregulated in aging muscle, and this is correlated with reduced expression of proteins involved in muscle biogenesis including IGF1, the p85α subunit of AKT, and the TF MYBL2. Overexpression of *miR‐29a/b* in muscle progenitor cells (MPCs) induces cell cycle arrest, possibly due to activation of both the p53/p21 and the p16 pathways, and reduces the expression of *IGF1*, *p85α*, and *MYBL2* via targeting of their 3′UTRs 47. An important connection made by this study is the interrelationship between Wnt signaling and *miR‐29a/b*‐mediated senescence, as stimulation of MPCs with Wnt3a activates the promoter of the *miR‐29a/b*. This may be relevant during normal aging‐induced sarcopenia, as systemic Wnt3a levels increase with age 48. Additionally, *miR‐29* is upregulated in primary fibroblasts undergoing OIS 31. Altogether, these reports suggest that miR‐29 could act as an age‐related senescence biomarker.

A recent study combining proteomic and *in silico* approaches in human senescent fibroblasts overexpressing *miR‐494* identified *HnRNPA3*, *RAD32B*, and *SYNCRIP* as differentially downregulated in these cells 49. The 3′UTR of these three mRNAs are targets for *miR‐494* as indicated by both luciferase and biotinylated‐miR‐494 pull‐down assays. Supporting a role for miR‐494 in repressing these genes in senescence, *HnRNPA3*, *RAD32B*, and *SYNCRIP* are downregulated in RS and DDR induced senescence. Inhibition of miR‐494 expression partially reverses the senescence phenotype and derepresses the expression of *HnRNPA3*, *RAD32B*, and *SYNCRIP*, an effect that is recapitulated by overexpression of constructs for these three genes lacking their 3′UTRs 49. Given the roles of these three proteins in RNA metabolism (HnRNPA3 and SYNCRIP; ref. 50), DNA repair (RAD32B; ref. 51), and telomere homeostasis (HnRNPA3; ref. 52), it is conceivable that their silencing by miR‐494 provides a favorable cellular milieu conducive for senescence onset on genomic or epigenomic insults.

Several studies have found a senescence‐promoting role for members of the miR‐34 family including *miR‐34* and *miR‐449*. In a small cohort of renal cell carcinoma (RCC) samples, miR‐34 was found to be consistently downregulated in tumors relative to surrounding tissue. When overexpressed in RCC lines, *miR‐34* causes a growth arrest that is in part mediated by direct targeting of the 3′UTR of *NOTCH1*, *E2F1*, *CDK6*, and *SIRT1* 53. In a mouse model of chronic lymphocytic leukemia, *miR‐34* is strongly upregulated at the leukemic stage of the disease. Its role in disease appears to be the attenuation of the proliferative capacity of leukemia cells via activation of the p53 tumor suppressor pathway 54. When used as the single parameter to assess disease progression in humans, the presence of *miR‐34* in leukemia cells is correlated with an increased disease‐free survival and reduced proliferation rates 54. Several experimental cancer models have indicated a senescence‐promoting role for *miR‐449*. Using microarray profiling in two mice models of gastric cancer (gastrin deficiency and *Helicobacter pylori* infection), *miR‐449* was identified as the only common downregulated miR. When overexpressed in a hepatocellular cancer cell line, *miR‐449* facilitates senescence by activating the p53/p21 tumor suppressor pathway as well as by targeting the 3′UTRs of *MYC*, *CDK6*, and *CCNE2* 55. Similarly, *miR‐449* activates an RB‐dependent senescence pathway in prostate cancer cells upon overexpression. The RB dependency appears to be indirect, as overexpression of *miR‐449* leads to RB dephosphorylation without evidence of targeting the 3′UTR of RB. In contrast, miR‐449 directly binds to the 3′UTR of the *CCND1* mRNA, thus providing a rationale for the growth arrest in these cells 56. In lung cancer cells, overexpression of *miR‐449* induces a senescence‐like growth arrest, and this is partially explained by reduced expression of the proliferative TF E2F3 57. Supporting this notion, miR‐449 targets the 3′UTR of *E2F3*. Altogether, the findings described by these studies may be clinically relevant as miR‐449 is often downregulated in lung, prostate, and gastric tumors.

### MiRs Regulating the SASP

Human fibroblasts engaging in senescence upregulate *miR‐146a* expression 58. Ectopic expression of *miR‐146a* in human fibroblasts undergoing senescence elicits a reduction in IRAK expression, a crucial receptor‐associated kinase that relays inflammatory signaling arising from interleukin 1 (IL1R) and Toll‐like receptors 59. Consequently, these cells feature impaired NF‐κB activation and reduced levels of key senescence‐reinforcing SASP cytokines such as IL6, IL8, and IL1α 12, 58. As the gene for *miR‐146a/b* is a target of the NF‐κB pathway and is upregulated during inflammatory responses 60, these data suggest a negative feedback loop model by which inflammatory responses are dampened by the miR‐146a/b‐dependent attenuation of the NF‐κB pathway at the receptor level.

An interesting study focused on the role of miR‐335 during senescence of human mesenchymal stem cells (hMSCs). In these cells, *miR‐335* expression is correlated with decreased proliferative potential induced by replication or cytokine stimulation 61. When overexpressed in hMSCs, *miR‐335* induces a p16‐dependent senescence phenotype characterized by decreased proliferation, SAHF formation, increased ROS generation, and induction of a SASP. Functionally, *miR‐335* blocks osteogenic differentiation potential of hMSCs *in vivo*, as *miR‐335* overexpressing hMSCs fail to form bone and cartilage when infused into the back of 8‐week‐old rats. hMSCs have protective immunomodulatory properties in several models of sepsis by attenuating the release of inflammatory cytokines via the polarization of macrophages to the alternative M2 state 62. Interestingly, mice injected with *miR‐335* overexpressing hMSCs display 30% survival after septic shock by lipopolysaccharide, whereas mice injected with normal hMSCs survive the challenge 61, possibly reflecting a defect in macrophage polarization by hMSCs. Thus, a senescence phenotype induced by *miR‐335* overexpression in hMSCs alters the specification of a macrophage subpopulation in this model. A key lesson from this study is that the interference of cell type‐specifying pathways by the induction of senescence can lead to the disruption of the biological function of a particular cell type.

In some instances, 3′UTRs of mRNAs are not only regulated by miRs but also by components of mRNA maturation process such as mRNA stabilization and splicing, with important implications for the stabilization of the corresponding protein and the downstream processes they mediate 63. Such a case has been recently described for C/EBPβ, a member of the C/EBP family of TFs (including C/EBPα and γ that play context‐dependent roles in the regulation of senescence through combinatorial heterodimerization; ref. 64). During OIS, C/EBPβ facilitates the establishment and maintenance of senescence by supporting the implementation of a SASP. Surprisingly, overexpression of a construct containing the 3′UTR of *C/EBPβ* in cells undergoing OIS restores proliferative capacity of these cells 65. Electrophoretic mobility shift assays of nuclei from these cells show decreased C/EBPβ DNA‐binding activity upon overexpression of its 3′UTR, and this is correlated with a lack of activating phosphorylation of C/EBPβ. This leads to a shift in the gene expression profile from an inflammatory to a proliferative one. Mechanistically, the RNA‐binding protein HuR binds to ARE elements in *C/EBPβ* 3′UTR and prevents its translocation to perinuclear sites, where mRNA export to the cytosol is initiated 66. This, consequently, results in reduced translation of C/EBPβ, thus favoring the formation of C/EBPα/γ heterodimers, which accounts for the aforementioned shift in transcriptional output 65. Altogether, these data indicate that nuclear retention of mRNAs encoding senescence regulators could be yet another elegant mechanism to modulate proliferative homeostasis and senescence.

### MiRs Regulating Transcription and Chromatin State During Senescence

The regulation of chromatin state by chromatin‐modifying activities and TFs is essential for transcription 67. An active chromatin promoter state is generally specified by a selection of modified histones including histone 3 trimethylated on lysine 4 (H3K4me3) and histone 3 acytelated on lysine 9 (H3K9ac), whereas an inactive chromatin state is distinguished by histone 3 trimethylated on lysine 27 (H3K27me3), monoubiquitination of histone H2A (H2AK119ub1), and histone 3 di/trimethylated on lysine 9 (H3K9me2/3). These modifications are effectuated by chromatin‐modifying enzymes such as PRCs PRC1 and PRC2 (mediating H2AK119ub1 and H3K27me3), SET domain containing and noncontaining HMTs (*e.g*., SUV39H and MLL, mediating lysine methylation), histone acetyl transferases (*e.g*., CBP/p300) mediating lysine acetylation, and histone demethylases (KDM1–6).

There is now accumulating evidence that miRs play an important role in the regulation of chromatin state and transcription through direct and indirect interactions with chromatin‐modifying activities and TFs 68 in cellular senescence. The retinoblastoma‐binding protein 2 (RBP2/JARID1A), a member of the KDM5 family of lysine demethylases, is negatively regulated by *miR‐212*. RBP2 preferentially catalyzes demethylation of active chromatin mark H3K4me3 to histone 3 dimethylated on lysine 4 (H3K4me2), and this coincides with decreased transcription of a subset of cytokines 69. In hepatocellular and gastric cancers, RBP2 is overexpressed, and this is inversely correlated to the expression of *miR‐212*. In HepG‐2 cells, downregulation of RBP2 or overexpression of miR‐212 leads to features of premature senescence as judged by increased levels of p16, p21, and p27 70. In contrast, overexpression of RBP2 favors anchorage‐independent growth. It is suggested here that RBP2 may promote senescence bypass by H3K4 demethylation at promoters of cell cycle inhibitors 70.

In a conditional *Pten* knockout mouse model for prostate cancer, a role for the TF ZBTB7a was recently reported 71. ZBTB7a is a transcriptional repressor that is differentially expressed in a variety of human cancers, in which it is thought to favor tumorigenesis by silencing of the *INK4‐ARF* locus through histone modification and chromatin remodeling 72. When, however, conditionally overexpressed in the prostate of *Pten*‐negative mice, Zbtb7a induces a senescence phenotype by activating the p53 tumor suppressor pathway. In contrast, *Zbtb7a* deletion in this tissue compromises the capacity of cells to undergo senescence, thus fostering invasive carcinoma. Transcriptional analyses of *Zbtb7a*‐negative prostates identified upregulation of the cancer‐promoting lncRNA H19, which is host to *miR‐675*, a miR that promotes proliferation by downregulation of RB 73. Chromatin immunoprecipitation (ChIP) analyses of *Zbtb7a*‐proficient and ‐deficient mouse prostate cells show occupancy of a Zbtb7a /Sox9 complex and Sox9, respectively, at the *H19* promoter. Based on these data, the authors propose that the function of Zbtb7a in prostate of *Pten*‐negative mice is to repress Sox9 transcriptional activity at the *H19* locus (and possibly other Sox9 target genes), probably by histone deacetylation. In its absence, Sox9 is able to recruit histone acetylase p300 to the *H19* promoter, leading to *H19* expression. Processing of *H19* into *miR‐675* is thought to underlie senescence bypass by destabilizing RB mRNA in prostate cancer cells 71.

In a recent study of OIS, ChIP followed by array hybridization (ChIP‐on‐chip) identified a common set of promoter targets shared by AGO2 and E2F family TFs in senescence 31. The promoters of AGO2/E2F target genes are characterized by features of transcriptionally inactive chromatin such as H3K9me3 and H3K27me3, and AGO2 preferentially colocalizes with these histone marks as well as with the histone variant macroH2A in senescent cells, a process now referred to as senescence‐associated TGS (SA‐TGS). Furthermore, AGO2 physically interacts with an RB corepressor complex, containing HDACs, at E2F target genes in senescent cells (Fig. 2). The specificity of this phenomenon is due to partial sequence complementarity between AGO2‐bound miRs and promoters, as exemplified by *miR‐let7* family‐dependent targeting of the AGO2/RB complex to a subset of promoters 31. Consistent with this notion, overexpression of AGO2 or *miR‐let7* in proliferating fibroblasts induces premature senescence, whereas their downregulation in cells undergoing senescence partially delays the establishment of senescence by impacting recruitment of RB to target genes. Thus, these data reveal an unexpected nuclear function for miRs in TGS of pro‐proliferative E2F target genes during senescence by establishing a transcriptionally repressive chromatin state at promoters.

## LncRNAs in the Modulation of Senescence

The lncRNAs are nonprotein coding transcripts that were arbitrarily defined as longer than 200 nucleotides in length to distinguish them from small regulatory RNAs like miRs, tRNAs, and so forth. LncRNAs are typically polyadenylated. Recent studies identified many different lncRNAs in the human genome by integrating numerous annotation sources in combination with RNA sequencing 19. They revealed several global properties of lncRNAs, including a tendency for location next to developmental regulators and enrichment of tissue‐specific expression patterns among others 74. Studies of lncRNAs overlapping promoter regions of protein‐coding genes identified numerous lncRNAs associated with cell cycle regulation, suggesting potential roles of lncRNA in biological processes and eventually in cell fate decisions 75. The past few years have also seen a growing number of examples for lncRNAs coordinating the access to or dissociation of regulatory proteins from chromatin. Several studies showed lncRNAs to recruit chromatin modifiers/remodelers to regulate transcription 76. Although many studies initially focused on lncRNAs associated with repressive chromatin‐modifying complexes, it is now also clear that active chromatin states are found associated with lncRNAs. Recently, genomic maps of lncRNA occupancy revealed principles of RNA–chromatin interactions, and the developed methodologies (ChIRP‐Seq and CHART‐Seq) pave the way to decipher intersections of RNA and chromatin with unprecedented precision genome wide 77, 78. Collectively, these studies demonstrate the critical importance of lncRNAs interfacing with chromatin‐modifying machineries to control chromatin states and gene activation. Despite their increasing biological relevance, the role of lncRNAs in senescence still remains fragmentary, with only a few examples described so far in the literature.

**Figure 3:**
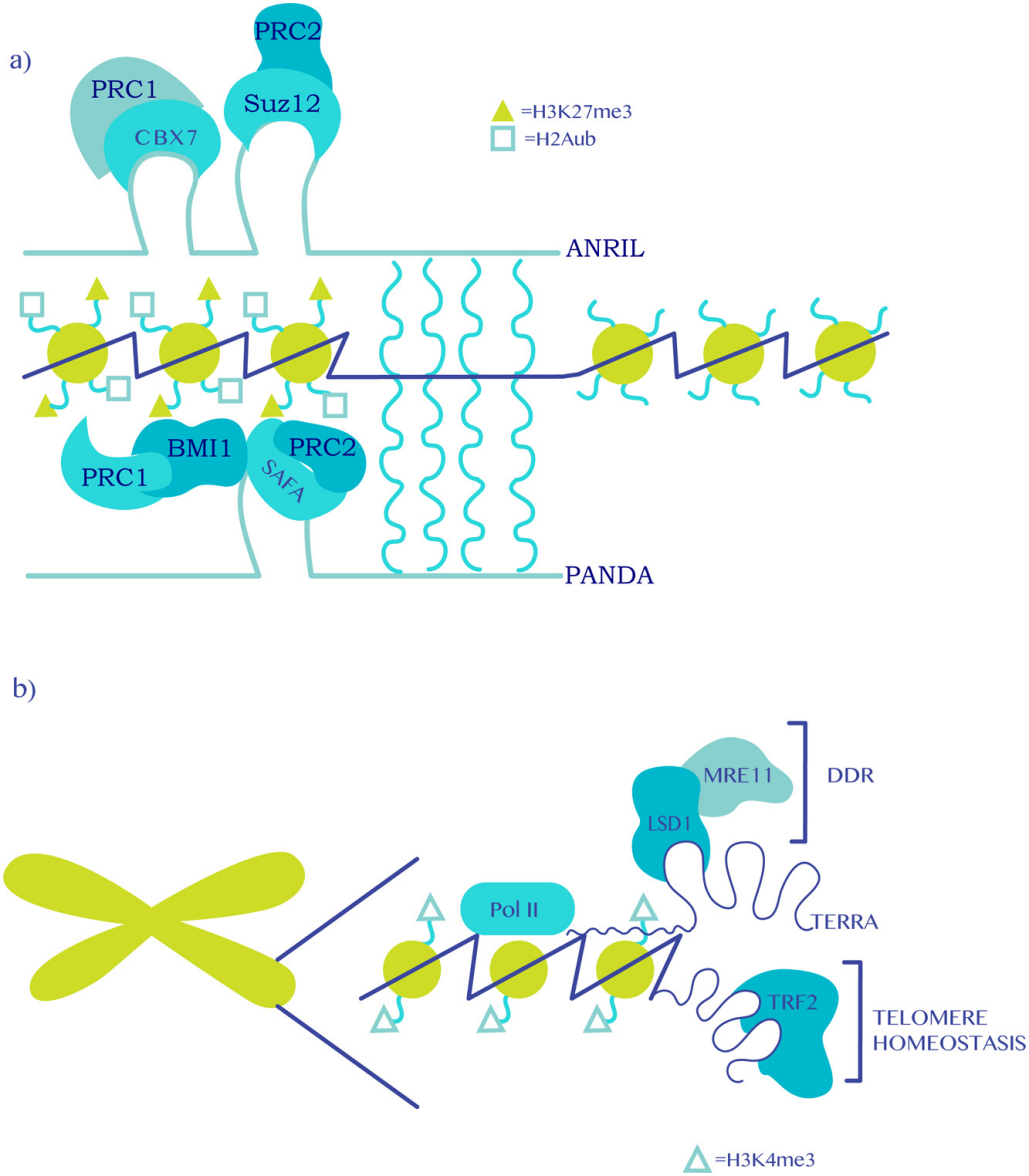
Senescence regulation by lncRNAs. (a) LncRNAs recruit PRC1 and PRC2 complexes to target promoters to modulate senescence via gene silencing. Recruitment may be direct operating in cis (*i.e*., CBX7 directly binds nascent ANRIL at the *INK4* locus) or indirect operating in trans via an adaptor molecule (*i.e*., PANDA‐mediated bridging between SAFA and BMI1 at target genes). (b) Noncoding transcription of TERRA at telomeres maintains telomere homeostasis by recruiting shelterin proteins (*e.g*., TRF2). In the absence of TRF2 (which occurs in replicative senescence due to telomere attrition), TERRA facilitates a DNA damage response (DDR) by recruiting an LSD1/MRE11 complex thus promoting senescence. Wavy blue lines indicate putative hybridization between lncRNA and a target promoter or other gene‐regulatory region.

Studies in primary human fibroblasts and prostate cancer cell lines dissected the role of lncRNA *ANRIL* (antisense ncRNA in the *INK4* locus) in the regulation of senescence 8, 9. *ANRIL* is a 3.8‐kb lncRNA that is transcribed in an antisense direction from the *INK4* locus 79. *ANRIL* associates with PRCs PRC1 and PRC2 through chromodomain proteins CBX7 and SUZ12 to repress the prosenescence *INK4* locus in prostate cancer cells and proliferating primary fibroblasts, thus suggesting an antisenescence function for the complex (Fig. 3a and Table 1). Indeed, when *ANRIL* is downregulated by overexpression of its corresponding antisense RNA in fibroblasts, the *INK4a/ARF/INK4B* locus is de‐repressed and senescence ensues. Under these conditions, the occupancy of *ANRIL*/PRC complexes at the *INK4* locus is reduced, pointing toward a recruiter role for *ANRIL* within the complex. Furthermore, recruitment of the complex to the *INK4* locus is dependent on RNA Pol II‐dependent transcription of the *INK4* locus itself, which presumably increases the local concentration of *ANRIL* to allow efficient CBX7‐mediated recruitment of PRC1. Silencing of the *INK4* locus may be reinforced by the binding of CBX7 to H3K27me3‐modified nucleosomes. Collectively, these data support a role of *ANRIL* in silencing of the *INK4* locus in cis through transcription‐dependent recruitment of CBX7/PRC1 and SUZ12/PRC2 complexes, possibly facilitating the formation of condensed chromatin configuration at the *INK4* locus. An open question is how the balance between sense and antisense transcription from the *INK4* locus is regulated. An attractive possibility is that a component of the repressive complex provides transcriptional directionality as previously shown for certain TFs 80.

A PRC‐dependent gene‐regulatory mechanism was also described for the p21‐associated ncRNA DNA damage activated (*PANDA*), which modulates senescence entry and exit through dynamic interactions with the transcriptional regulator “scaffold attachment factor” (SAFA alias hnRNPU) as well as PRC1 and PRC2 complexes, however, in contrast to ANRIL in trans and not in cis (Fig. 3a and Table 1; ref. 81). SAFA is a chromatin‐associated protein with RNA‐ and DNA‐binding activities that has been previously involved in promoting the deposition of H3K27me3 and *Xist* RNA at the inactive X chromosome (Xi; ref. 82). Consistent with this, SAFA and PRC complexes physically colocalize at a subset of PRC prosenescence target genes (*e.g*., p21, IL8, and BMP2), and silencing of SAFA (which induces senescence) or induction of senescence (where SAFA becomes downregulated) causes the derepression of prosenescence PRC target genes, an event that is paralleled by reduced occupancy of the SAFA‐containing PRC complexes and loss of H3K27me3 and H2AK119ub1 at the respective promoters. These data, thus, imply that SAFA facilitates the recruitment of PRCs to their respective targets genes. Further complexity is added by the dynamic expression of *PANDA* during the establishment and maintenance of senescence. In proliferating fibroblasts, *PANDA* is essential in mediating SAFA–PRC1 interactions, possibly by acting as an adaptor between SAFA and BMI1, and is thus key to the repression of prosenescent genes (including its own expression). However, in senescent cells, where SAFA expression is downregulated and *PANDA* is upregulated, *PANDA* is now found to reinforce the senescent phenotype by decoying TF NF‐YA, which collaborates with E2F TFs in proliferating cells to activate the expression of pro‐proliferative genes 83. Thus, this study portrays the dynamic role of lncRNA *PANDA* in the maintenance of cell identity. Indeed, the multimodular nature of *PANDA* allows it to dynamically respond to different cellular cues (*i.e*., proliferative versus senescence‐inducing stimuli) by interfacing with multiple chromatin‐modifying activities and TFs to regulate gene expression 81. It thus acts like a molecular “chameleon.” Importantly, this mechanism may be broadly applicable to other lncRNAs driving cell fate decisions.

LncRNAs were also shown to modulate senescence by interacting with proteins involved in RNA metabolism. An example of this mode of action was recently described for the HOX Antisense Intergenic RNA (*HOTAIR*) in human cancer cells. *HOTAIR* was originally found to silence the *HoxD* locus; however, it is now known to target many different loci for PRC2‐dependent silencing 84. *HOTAIR* plays a role in regulating the half‐life of RNA‐stabilizing proteins via a dynamic interplay with the RNAi and ubiquitination machineries 85. In HeLa cells, a trimeric complex containing AGO2, miR‐let7, and RNA‐binding protein HuR promote *HOTAIR* decay. In a separate complex, *HOTAIR* interacts with the E3 ubiquitin ligases DZIP3 and MEX3 as well as the RNA‐binding proteins ATXN1 and SNUPN (*alias* Snurportin). When the stability of *HOTAIR* is enhanced either by its overexpression or by downregulation of HuR, ATXN1 and SNUPN are degraded via the proteasome pathway through their ubiquitination by DZIP3 and MEX3. This mechanism is supported by *in vitro* ubiquitination assays, which show that the complex facilitates the ubiquitination of ATXN1 and Snurportin. Interestingly, in senescent cells, where the expression of *HOTAIR* is augmented, ATXN1 and Snurportin are highly ubiquitinated, suggesting that their degradation might facilitate senescence. Indeed, downregulation of *HOTAIR* impacts senescence execution, and this is correlated with increased stability of ATXN1 and Snurportin 85. Although the function of ATXN1 and SNUPN in senescence as well as the mechanisms leading to the stabilization of *HOTAIR* in senescent cells are not clear, this study supports a dynamic interplay by which competition for *HOTAIR* binding either to a HuR/AGO2 or DZIP3/MEX3 E3 ligase complex regulates the stability of the ATXN1 and SNUPN.

LncRNAs have also been shown to regulate senescence through the maintenance of telomere homeostasis. This is exemplified by the telomeric repeat‐containing RNAs (*TERRA*), a family of ncRNAs of variable lengths that are transcribed from subtelomeric regions. *TERRA* is highly conserved among species, and its expression is regulated by the RNAi and nonsense‐mediated decay pathways. *TERRA* transcription is correlated with H3K4me3 presence at telomeres, both of which decrease during senescence 86. It has been hypothesized that *TERRA* maintains telomere homeostasis by recruiting shelterin proteins to telomeres in a transcription‐dependent manner (Fig. 3b and Table 1; ref. 87). On dysregulation of shelterin proteins, *TERRA* has been found to promote the recruitment of the histone demethylase LSD1 to telomeres, facilitating a telomeric DDR via an interaction between LSD1 and MRE11 (Fig. 3b and Table 1; ref. 88). It is tempting to speculate that a similar mechanism occurs during RS, where telomere attrition incurs a chronic DDR. Additionally, it has been suggested that G‐quadruplex structures within *TERRA* induce cellular senescence, whereas telomeric *DNA‐TERRA*‐G‐quadruplex heteroduplexes promote bypass of senescence 89. Thus, the recruitment of telomere‐associated factors can be regulated by different *TERRA*‐containing nucleic acid species in cells. A key point arising from these data is that the dynamics of *TERRA* transcription during both normal aging and on senescence‐inducing stimuli, particularly after DNA damage, is key to determining cell fate. Indeed, accelerated rates of *TERRA* transcription may support proliferation at the expense of a chronic telomeric DDR, which on accumulation of DNA damage or normal age‐related telomere attrition would lead to an eventual halt in *TERRA* expression that can contribute to the onset and establishment of senescence.

## Perspectives on the Role of miRs and lncRNAs in Senescence

Despite increasing efforts, our knowledge with regards to ncRNA functions in senescence must still be considered fragmentary. To gain a deeper understanding of the roles miRs and lncRNAs play in senescence, several key issues need to be addressed. For instance, the regulation of miR and lncRNA gene expression during senescence onset and/or bypass remains poorly understood. What is the role of AGO proteins other than AGO2 in senescence? What are the stimuli required to activate or repress ncRNA genes? What mechanisms underlie the specificity of ncRNA transcriptional regulation during senescence? Which are the main TFs and associated chromatin‐modifying activities that regulate ncRNA transcription in this context? Is there a common set of TFs and chromatin‐modifying activities to all types of senescence‐inducing stimuli or do they differ depending on stimulus and cell type? To address these questions and integrate the available data, a system‐based approach is required. To unravel the regulatory principles underlying ncRNA expression and transcriptional regulation of target genes during senescence onset and/or bypass, temporal analyses of ncRNA expression need to be performed. The generation of such dynamic profiles can be used as input for bioinformatics analyses to generate predictions regarding the transcriptional regulators of and the genomic targets for miRs and lncRNAs at specific time points during senescence onset and/or bypass, possibly identifying the transcriptional paths required to reach the senescent phenotype. The identification of such transcriptional regulators and expression paths may provide opportunities for the modulation of the senescent transcriptome. Along these lines, an additional issue that remains to be addressed is that of regulation of RNA Pol II at ncRNA genes, including elongation and directionality of transcription. Given the pervasive nature of noncoding transcription in cells 90, how can transcription be dynamically regulated to achieve the specificity required for the induction of the ncRNA transcriptome that facilitates the instigation, maintenance of, and/or exit from the senescent phenotype? For instance, both *ANRIL* and *PANDA*, which can promote senescence bypass or exit 8, 81, are expressed in an antisense direction from the *INK4* and *CDKN1A* loci, both of which code for key senescence effectors (*i.e*., p16 and p21). Thus, depending on the directionality of Pol II transcription at these loci (*i.e*., coding versus noncoding transcription), the cell is able to make a choice to whether enter or bypass/exit senescence. How the cell arrives to this decision is presently not known. To shed light on this issue, temporal RNA‐seq and Pol II ChIP‐seq analyses during senescence onset and/or bypass are required to determine the distribution of Pol II at key loci and its correlation with coding and noncoding transcription. Furthermore, it is conceivable that the intra (*i.e*., DDR‐associated) or extracellular (*i.e*., SASP) signaling arising from senescence onset and/or bypass may regulate the formation of a mature RNA Pol II complex able to engage in productive transcription (*i.e*., via activation of P‐TEFb‐mediated phosphorylation of serine 2 of Pol II C‐terminal domain). In this respect, it would be important to evaluate the role of DDRNAs/diRNAs in the regulation of ncRNA expression, as they have been shown to recruit chromatin‐modifying complexes at sites of damage in a transcription‐dependent manner 21, 22. This possibility is likely non‐negligible as a chronic DDR is a feature of the senescent cell 22 and may therefore represent a common pathway to different types of senescence‐inducing stimuli. Another interesting possibility that remains to be explored pertains to the propagation of senescence‐inducing and/or ‐bypassing by miR‐containing exosomes as part of the SASP 91. This may be relevant in the *in vivo* setting, where components of the SASP (possibly including miRs) may provide transcriptional specificity to drive the senescence phenotype in a particular tissue. In summary, the mechanisms underlying the transcriptional specificity of ncRNA genes in cellular senescence remain to be determined.

Like any other cell type, senescence is arrived at after at least a few cell divisions during which the cell must be able to successively transmit the information received from the initial senescence‐inducing stimuli to the next generation until the maintenance phase of senescence. This information is likely stored/memorized at the chromatin level and read‐out in the transcriptional output. ncRNAs may be candidate molecules that can transmit this information. Indeed, during OIS, a miR/AGO2/RB/HDAC corepressor complex is assembled to repress pro‐proliferation E2F target genes by implementing a repressive chromatin state specified by H3K9me3 and H3K27me3 31, although a complete account of involved chromatin‐modifying entities remains to be established (Fig. 2). As both H3K9me3 and H3K27me3 have been shown to maintain and propagate silent chromatin states by the recruitment of HP1/SUV391 and PRC complexes, respectively 92, 93, it would be interesting to establish a correlation between noncoding transcription at the promoters of E2F target genes, AGO2/RB HDAC complex recruitment, deposition of repressive chromatin marks, and expression of E2F target genes across cell divisions on the onset and maintenance of senescence. Indeed, the possibility of propagation of silent chromatin states of proliferative genes during senescence is likely, at least for PRC2‐silenced genes, as the PRC2 complex can remain bound to chromatin after replication 94. Despite the apparent rigidity of the senescent phenotype, disruption of the p53/p21 and/or RB/p16 pathways can lead to senescence exit, demonstrating a certain degree of plasticity within the senescent phenotype. To this effect, senescent cells must be able to reawaken the expression of pro‐proliferation genes and reciprocally silence senescence effector genes, a process that naturally involves the modification of chromatin and its subsequent transmission to the next cell generation. In this context, lncRNAs have been shown to facilitate this transition 81, although it remains unclear whether they are required to preserve the proliferative state after senescence exit. Additionally, the signal transduction pathways promoting cytonuclear shuttling of lncRNAs and miRs and their subsequent nuclear retention/exclusion during senescence remain to be identified. An indirect role for IPO8 in the nuclear localization of AGO2 in mammalian cells has been described recently 27, although a direct interaction between these two proteins remains to be established. By contrast, how differential export of ncRNAs is regulated is currently completely unknown. Furthermore, a 5‐base motif (AGCCC) within the lncRNA *BORG* and a 6‐base motif (AGUGUU) in the 3′ terminus of *miR‐29b* were found to mediate their nuclear localization 95, 96. Systematic computational and structural studies are required to identify structurally similar motifs in lncRNAs and miRs involved in the regulation of senescence. In brief, the roles of both miRs and lncRNAs in the propagation of senescence‐inducing, ‐bypassing, and ‐exiting signals need to be studied in more detail and more systematically.

## Conclusion

The foregoing discussion has outlined the current state‐of‐affairs regarding the role of ncRNAs in senescence. Accumulating evidence clearly shows that both miRs and lncRNAs play important roles in the modulation of the senescent phenotype by regulating gene expression at the transcriptional and post‐transcriptional levels as well as by promoting telomere homeostasis and repair. However, our current knowledge pertaining to the role of ncRNA in senescence still remains rather rudimentary. Dedicated efforts are required to establish general principles for the roles of ncRNA in cellular senescence. These efforts are necessary to expand our understanding of the biological function of senescence in (patho)physiology.
